# Evaluation of the relationship between the biosecurity status, production parameters, herd characteristics and antimicrobial usage in farrow-to-finish pig production in four EU countries

**DOI:** 10.1186/s40813-016-0028-z

**Published:** 2016-05-01

**Authors:** Merel Postma, Annette Backhans, Lucie Collineau, Svenja Loesken, Marie Sjölund, Catherine Belloc, Ulf Emanuelson, Elisabeth grosse Beilage, Elisabeth Okholm Nielsen, Katharina D. C. Stärk, Jeroen Dewulf, on behalf of the MINAPIG consortium

**Affiliations:** 1 Veterinary Epidemiology Unit, Department of Reproduction, Obstetrics and Herd Health, Faculty of Veterinary Medicine, Ghent University, Salisburylaan 133, 9820 Merelbeke, Belgium; 2 Department of Animal Health and Antimicrobial Strategies, National Veterinary Institute, SVA, SE-751 89 Uppsala, Sweden; 3 Department of Clinical Sciences, Swedish University of Agricultural Sciences, P.O. Box 7054, SE-750 07 Uppsala, Sweden; 4 SAFOSO AG, Waldeggstrasse 1, CH-3097 Liebefeld, Switzerland; 5 UMR1300 BioEpAR, LUNAM Université, Oniris, INRA, BP40706, F-44307 Nantes, France; 6 Field Station for Epidemiology, University of Veterinary Medicine Hannover, Büscheler Straße 9, D-49456 Bakum, Germany; 7 Danish Agriculture and Food Council, Axeltorv 3, DK-1609 Copenhagen V, Denmark

**Keywords:** Antimicrobial usage, Biosecurity, Production parameters, Pig production, Causal path

## Abstract

**Background:**

High antimicrobial usage and the threat of antimicrobial resistance highlighted the need for reduced antimicrobial usage in pig production. Prevention of disease however, is necessary to obtain a reduced need for antimicrobial treatment. This study aimed at assessing possible associations between the biosecurity level, antimicrobial usage and farm and production characteristics in order to advice on best practices for a low antimicrobial usage and maximum animal health and production.

A cross-sectional study was conducted in 227 farrow-to-finish pig herds in Belgium, France, Germany and Sweden between December 2012 and December 2013. Associations between biosecurity status, antimicrobial usage, and production parameters were evaluated with multivariable general linear models, according to an assumed causal pathway.

**Results:**

The results showed that higher antimicrobial usage in sows tended to be associated with higher antimicrobial usage from birth until slaughter (*p* = 0.06). The antimicrobial usage from birth until slaughter was positively associated with the number of pathogens vaccinated against (*p* < 0.01). A shorter farrowing rhythm (*p* < 0.01) and a younger weaning age (*p* = 0.06) tended to be also associated with a higher antimicrobial usage from birth until slaughter whereas a better external biosecurity (*p* < 0.01) was related with a lower antimicrobial usage from birth until slaughter.

**Conclusion:**

Management practices such as weaning age and biosecurity measures may be important factors indirectly impacting on antimicrobial usage. We therefore promote a holistic approach when assessing the potential to reduce the need for antimicrobial treatments.

## Background

In many countries of the European Union (EU) pig production is amongst one of the highest using sectors of antimicrobial (AM) agents in animal production as reported in detail for some EU countries [1–3]. After the discovery of penicillin by Fleming in 1928 and its subsequent usage around world war II antimicrobials became very important in the curing of bacterial infections in both humans and animals. Unfortunately however, bacteria are capable of developing resistance mechanisms against the antimicrobials used, either by genetic mutations or by taking up resistance genes from other bacteria [4]. This resistance selection is mainly triggered by the use of antimicrobials (Callens B.F., Boyen F., Berge A.C., Chantziaras. I., Haesebrouck F., Dewulf J., Epidemiology of acquired antimicrobial resistance in bacteria from food-producing animals, submitted). EU countries with a high antimicrobial usage (AMU) rank also high in their resistance levels [5]. Therefore, reduced and prudent antimicrobial usage in animals became of high interest in recent years, mainly due to the public health threat of antimicrobial resistance (AMR) development and possible transmission from the animal to the human population [6-9]. The first efforts in some EU countries show that a reduced usage of antimicrobials results in reduction of resistance levels as well [3, 10], which is the main focus of the international fight against antimicrobial resistance in animal production [11].

To be able to reduce antimicrobial usage, it is important to ensure healthier animals and therefore reduce the necessity for antimicrobial treatment. Some authors have suggested a broad range of possible alternatives [12–14], for example the increased use of vaccines to make animals less sensitive to infections [15–18] or an improved management and increased biosecurity level [19, 20]. However, several of these suggested alternatives are based upon clinical observations or rational deduction rather than quantitative observations making them prone to critics due to insufficient scientific bases of their efficacy for the replacement of antimicrobials.

Therefore, a good insight in the associations between preventive measures, management factors, production parameters, biosecurity status and antimicrobial usage is of critical importance to better understand the value of the different alternatives and to help herd advisors and farmers in the optimization of their farm management. Knowing whether such associations exist provides researchers, farmers, herd advisors (e.g. veterinarian, feed advisor, climate specialist) and policy makers with potential tools to improve herd production combined with reduced necessity of antimicrobial products.

This study aimed at studying and visualizing associations between management characteristics, production parameters, biosecurity status and antimicrobial usage data from four EU countries. The results of this study will be used by the MINAPIG consortium to study the implementation of high biosecurity, vaccines and herd health management measures as potential drivers for reduced antimicrobial usage in pig production.

## Methods

### Herd selection

This study was performed in four EU countries with a medium to highly intensive pig production [21]; Belgium, France, Germany and Sweden. Per country the aim was to include 60 farrow-to-finish herds with ≥ 100 sows and ≥ 500 finishers. For Belgium an email list of pig farmers who subscribed to a newsletter issued by the faculty of veterinary medicine of Ghent University was used to select the herds based on willingness to participate. Only the Dutch speaking part of Belgium, Flanders, which represents 90 % of pig production in Belgium [22], was included in the study due to logistic reasons. Herds in the north-western part of France, representing 75 % of the country’s pig production, were randomly selected from a database of the Institute for pig and pork industry. In Germany the herds were selected from consultancy circles and with veterinarians’ input in the three regions with the largest pig production, Niedersachsen, Nordrhein-Westfalen and Mecklenburg-Vorpommern (64 % of total German production) [23]. A request for participation by their herd veterinarian or a consortium partner was used to reach the 60 participating pig farmers in Sweden.

Finally in Belgium 52 herds participated in this retrospective study and in the other three countries there were 60 participants. For five Belgian herds the information on the antimicrobial usage was not complete, resulting in a total of 47 herds used in the analyses for Belgium and a total of 227 herds in the study. Our criterion of including herds with ≥100 sows had to be lowered to ≥70 sows to reach the maximum of participating herds. Three Belgian herds, six French herds and one Swedish herd had a number of sows between 70 and 100.

### Herd visit

A strict protocol was used to visit and interview the participating herds, guaranteeing a similar collection and entry of data over the countries. Interviewers received a training to standardize the method for data collection. Furthermore, the participating herds were visited by one veterinarian/researcher in Belgium, one in France and one in Germany and by two veterinarians/researchers or a veterinarian from the Swedish Animal Health Service (*n* = 15) in Sweden. Agreement between the project partners on the completeness and accuracy of the herd visit protocol was reached by consultation, discussion and consensus.

Herds were visited once on a convenient day in the period between December 2012 and December 2013. A farm inspection in combination with the completion of the questionnaire was performed by the interviewer during the herd visit. The collected herd management and technical parameter information corresponded to the year preceding the herd visit.

### Data collection

Technical parameters (e.g. number of weaned piglets per sow per year (WSY)) and herd management characteristics (e.g. farrowing rhythm) were collected, together with information on the biosecurity status of the herd using the risk-based scoring system Biocheck.UGent™ (www.Biocheck.UGent.be). The technical parameters were collected from the herd management system if available or by interviewing the farmer.

The farrowing rhythm refers to the interval, expressed in weeks, between the birth of two batches of piglets. In this study this ranged between a 1-week system and a 5-week system for Belgium, France and Germany, while in Sweden systems with a farrowing rhythm of over 5-weeks were also used. The latter were coded for analysis as >5-week systems. The number of weaned piglets per sow per year was calculated as the number of litters per year times the number of liveborn piglets per sow minus the mortality until weaning. The weaning age was expressed as the average duration, in days, from the birth of a piglet until it was weaned. The number of pathogens vaccinated against was created by summing up all vaccinations used in a herd, either for sows, boars, gilts or piglets on the date of the herd visit, except the vaccine used for immune-castration of male animals. For combination vaccines every single pathogen they have activity against was accounted for separately. Anti-inflammatory, anti-coccidial and zinc-oxide usage was expressed as being applied yes or no. A more detailed description of the other variables mentioned in Table 1, such as the gender and education level, can be found in [19]. The questionnaire can be obtained upon request from the first author.

**Table 1:** Results of univariable and multivariable general linear regression models

			Country corrected univariable			Country corrected multivariable	
Outcome variable	Risk factor	N	β-coefficient	*p*-value	Adjusted R^2^	β-coefficient	*p*-value
LOG TI Breeding	TI 200	227	<0.01	<0.01	0.148	<0.01	**0.01**
	Internal biosecurity	227	0.22	0.36	0.073		
	External biosecurity	227	0.51	0.08	0.083		
	Years experience	221	−0.07	0.81	0.071		
	Pathogens vaccinated	227	2.35	0.14	0.079		
	# sows	227	0.01	0.33	0.074		
	# employees	221	0.43	0.78	0.066		
	Gender	214		0.60	0.071		
	Male	137	3.14	0.60			
	Female	77	Ref.	Ref.			
	Education	210		0.11	0.082		
	Lower	84	−15.47	0.05			
	Higher	84	−15.77	0.06			
	University	42	Ref.	Ref.			
	Farrowing rhythm (cat)	219		0.76	0.060		
	>5	18	−0.81	0.95			
	5	20	2.76	0.82			
	4	48	−1.34	0.89			
	3	80	4.33	0.62			
	2	21	14.94	0.20			
	1	32	Ref.	Ref.			
LOG TI 200	TI Breeding	227	<0.01	**<0.01**	0.332	<0.01	**<0.01**
	Internal biosecurity	227	−0.01	0.11	0.325		
	External biosecurity	227	−0.02	**0.01**	0.353	−0.03	**<0.01**
	Weaning age	216	−0.05	**0.05**	0.335	−0.05	0.06
	Years experience	221	<0.01	0.28	0.324		
	Pathogens vaccinated	227	0.18	**<0.01**	0.355	0.14	**<0.01**
	# sows	227	<0.01	**0.01**	0.346		
	# employees	221	0.05	0.32	0.315		
	Gender	214		0.51	0.313		
	Male	137	0.11	0.51			
	Female	77	Ref.	Ref.			
	Education	210		0.39	0.331		
	Lower	84	0.07	0.77			
	Higher	84	0.28	0.24			
	University	42	Ref.	Ref.			
	Zinc oxide	205		0.29	0.310		
	Yes	39	0.25	0.29			
	No	166	Ref.	Ref.			
	Anti-inflammatory weaners	227		**0.05**	0.338		
	Yes	71	0.37	0.05			
	No	156	Ref.	Ref.			
	Anti-coccidial	214		0.10	0.313		
	Yes	90	0.28	0.10			
	No	124	Ref.	Ref.			
	Farrowing rhythm	219		**<0.01**	0.360		**<0.01**
	>5	18	−0.78	0.05		−0.88	**0.02**
	5	20	−1.15	<0.01		−1.10	**<0.01**
	4	48	−0.51	0.07		−0.44	0.11
	3	80	−0.38	0.12		−0.21	0.42
	2	21	0.10	0.75		−0.06	0.85
	1	32	Ref.	Ref.		Ref.	Ref.
Number of weaned piglets per sow per year (WSY)	Years experience	217	−0.02	0.27	0.354		
	External biosecurity	223	0.05	**<0.01**	0.362		
	Internal biosecurity	223	0.03	0.06	0.349		
	Weaning age	212	−0.17	**<0.01**	0.367	−0.19	**<0.01**
	Pathogens vaccinated	223	0.18	0.06	0.349		
	Mortality until weaning	222	−0.18	**<0.01**	0.423	−0.21	**<0.01**
	#sows	223	<0.01	**<0.01**	0.391		
	#employees	217	0.22	**0.02**	0.359		
	TI Breeding	223	0.01	**<0.01**	0.362	0.01	0.06
	TI 200	223	<0.01	0.71	0.339		
	Anti-inflammatory sucklers	223					
	Yes	122	0.43	0.39	0.340		
	No	105	Ref.	Ref.			
	Anti-inflammatory weaners	223					
	Yes	71	0.57	0.13	0.345		
	No	156	Ref.	Ref.			
	Anti-inflammatory sows	215					
	Yes	217	1.18	0.49	0.343		
	No	2	Ref.	Ref.			
	Anti-coccidial	211					
	Yes	90	0.08	0.82	0.337		
	No	124	Ref.	Ref.			
	Zinc oxide	201					
	Yes	39	0.86	0.06	0.363		
	No	166	Ref.	Ref.			
	Country * Weaning age						**0.02**
	Belgium * Weaning age					−0.20	0.18
	France * Weaning age					0.21	**0.04**
	Germany * Weaning age					0.15	0.18
	Sweden * Weaning age					Ref.	Ref.

*LOG* log transformation. Light gray values in the univariable model indicate that these factors were not significant (*p* < 0.20) in the univariable model. In the multivariable model p-values which are significant with *p* < 0.05 are black and bold, 0.05 < *p* < 0.10 are black and *p* > 0.10 are light gray. Significant interactions are listed where applicable. All models were corrected for the country effect by adding country in the model as a fixed variable. Only relevant variables are listed

### Biosecurity quantification

The biosecurity status of the participating farms was quantified by using the risk-based tool Biocheck.UGent™ [24]. This assessment tool makes comparison of the biosecurity status of herds within and between countries possible by returning 109, mainly dichotomous and trichotomous, questions into a score from 0 to 100 for both external and internal biosecurity, where zero means absolute lack of any biosecurity measures and 100 means declaration of full application of all assessed biosecurity measures. The Biocheck.UGent™ consists of 6 subcategories for internal biosecurity (1. disease management, 2. farrowing and suckling period, 3. nursing unit, 4. fattening unit, 5. measures between compartments, 6. working lines) and 6 for external biosecurity (1. purchase of breeding pigs, 2. purchase of piglets, 3. artificial insemination, 4. transport of animals, 5. feed and water supply, 6. removal of manure and dead animals). The Biocheck.UGent™ system is described in more detail in Laanen et al. [20, 25], Backhans et al. [26] and Postma et al. [19] in which it has shown to be a comprehensive, repeatable scoring system with a predictive and discriminating validity.

### Antimicrobial usage quantification

Information on the antimicrobial usage for the preceding year in Belgium, Germany and Sweden, and the last batch in France, was collected at in point in time. Invoices from the veterinarian and feed company combined with information from the farmer were used in Belgium. In France this information came from the journal of treatment of and interview with the farmer. While in Germany the delivery and treatment forms from the prescribing veterinarian were used. In Sweden paper copies derived from treatment records, which are mandatory and inspected by the county administration board, were used.

From the collected information the product name including details such as formulation and concentration, amount purchased/used and the animal category in which it was used were registered. If the animal category in which the product was used was not explicitly mentioned on the invoice, the farmer was asked to provide more information.

Herd level antimicrobial usage data were used to calculate the “treatment incidence” (TI) per herd and age category by the formula described below and as described and used before in several publications [1, 20, 27, 28].

$$ \mathrm{T}\mathrm{I}=\frac{\mathrm{Total}\ \mathrm{amount}\ \mathrm{of}\ \mathrm{active}\;\mathrm{substance}\ \mathrm{administered}\ \left(\mathrm{mg}\right)}{\mathrm{DDDA}\ \left(\frac{\mathrm{mg}}{\mathrm{kg}}\right)\kern0.5em *\kern0.5em \mathrm{number}\ \mathrm{of}\ \mathrm{days}\ \mathrm{at}\ \mathrm{risk}*\mathrm{kg}\ \mathrm{animal}\ \mathrm{at}\ \mathrm{risk}}\kern0.5em *\kern0.5em 1000\ \mathrm{pigs}\ \mathrm{at}\ \mathrm{risk} $$

The TI is a technical unit of measurement that quantifies how many animals out of a theoretical group of 1000 animals received daily an AM treatment. Or, if one animal would live for a theoretical period of 1000 days, how many of these days it would have been treated with an antimicrobial. Divided by 10 this gives the percentage of the lifespan an average animal on this herd was treated with a daily dose of antimicrobials. Combined TI’s were calculated for sows, gilts and boars (TI Breeding) and over a standardized period at risk of 200 days for the lifespan of a pig from birth until slaughter (TI 200). The 200 days, as the standard duration between birth and slaughter, was agreed upon based on consensus between the project partners from the participating countries. This TI 200 days makes it easier to compare the usage over countries, since the period at risk is standardized between these countries. For sows the period at risk was set to 1 year.

To be able to compare the usage over countries a standardised assumed weight at treatment was set for the different age categories; suckling piglet = 2 kg, weaner = 7 kg, finisher = 35 kg, sow = 220 kg. Furthermore, to be able to compare the different products and their concentrations within similar antimicrobial classes used in the different countries, a consensus defined daily dose animal (DDDA) per antimicrobial class, including consensus long acting factors, were established. The procedure used to come to these consensus DDDAs was extensively described in Postma et al. [29].

### Data processing

A LOG transformation of the data for the number of sows as an outcome variable (data not shown) in the regression models was needed to correct for the right skewedness of this variable.

Outcomes for TI 200 and TI breeding were also LOG transformed using SPSS statistics 22 (IBM), after adding one to the original outcome to adjust for zero values in the data.

Biocheck.UGent™ is a webbased scoring system using Limesurvey.

### Statistical analysis

Initially all possible causal routes linking antimicrobial usage, biosecurity status, herd characteristics and technical parameters (e.g. number of sows, WSY, average daily weigh gain (ADG, g/day), mortalities (%)) were identified based on logical reasoning with the main focus on parameters influencing the antimicrobial usage or the ones being influenced by the antimicrobial usage. Subsequently each of the identified possible associations was assessed using a regression model with the specific predictor always in combination with country as a second predictor variable to account for country specific characteristics.

All associations that showed a univariable p-value of < 0.20 were retained for the multivariable analysis. The multivariable general linear model was constructed using the stepwise backward selection procedure, including testing of two-way interactions of significant main effects. Confounding effects were evaluated during the modelling process by checking changes in parameter estimates. The association in the multivariable linear regression model was considered significant if *p* < 0.05 and a p-value between 0.05 and 0.10 was considered nearly significant and relevant to describe. Normal probability tests and plots were examined to check whether the assumptions of normality and homoscedasticity of residuals were fulfilled.

All statistical analyses were performed using SPSS statistics 22 (IBM). All tested variables can be found in Table 1.

## Results

### Farm characteristics

A 3-week farrowing rhythm system was most commonly used (80/227 herds). Followed by a 4 week system (48/227), a 1-week system (32/227), a 2-week system (21/227), a 5-week system (20/227) and a >5-week system (18/227). The mean weaning age was highest in Sweden (35 days) and lowest in Belgium (23.5 days). The mean number of piglets weaned per sow per year was comparable in Belgium (27.2, SD = 2.6), France (26.5, SD = 2.3) and Germany (27.4, SD = 2.3), but lower in Sweden (23.2, SD = 2.3). In Belgium, France and Germany the number of pathogens vaccinated against had a median of 7, while in Sweden this was 4. Out of 227 herds, 71 reported to use anti-inflammatory products in the weaners, while 90 out of 227 used anti-coccidial products in the suckling piglets.

Other herd characteristics of interest were described in more detail in the publication of Postma et al. [19].

### Biosecurity status

The external biosecurity level (65.5, range 43–93) was overall higher compared to the internal biosecurity level (55.7, range 6–88). External biosecurity was highest in Germany (70.2) and lowest in France (59.4), while the internal biosecurity level was highest in Sweden (58.8) and lowest in Belgium (50.3). In Postma et al. [19] results of the biosecurity quantification in the herds in the four participating countries and the link with production characteristics were described in detail. Since five Belgian herds were lacking information on antimicrobial usage they were removed from analysis in this study, resulting in slightly different results compared to the ones published in Postma et al. [19].

### Antimicrobial usage

Average antimicrobial usage in the breeding animals (23.0) was lower compared to the usage from birth until slaughter (TI 200) in the growing pigs (128.3). For both the TI 200 days (Sweden = 22.7; Germany = 242.8) and the TI breeding animals (Sweden = 10.9, Germany = 42.0) Sweden was the lowest using country and Germany the highest.

The quantification of the antimicrobial usage and the results in the four countries is described in detail in Sjölund et al. (Sjölund M., Postma M., Collineau L., Lösken S., Backhans A., Belloc C., Emanuelson, U., Groβe Beilage, E., Stärk, K. D. C., Dewulf, J., Quantitative and qualitative antimicrobial usage patterns in farrow-to-finish pig herds in Belgium, France, Germany and Sweden, submitted).

### Associations between antimicrobial usage, biosecurity level and farm characteristics

**Fig. 1:**
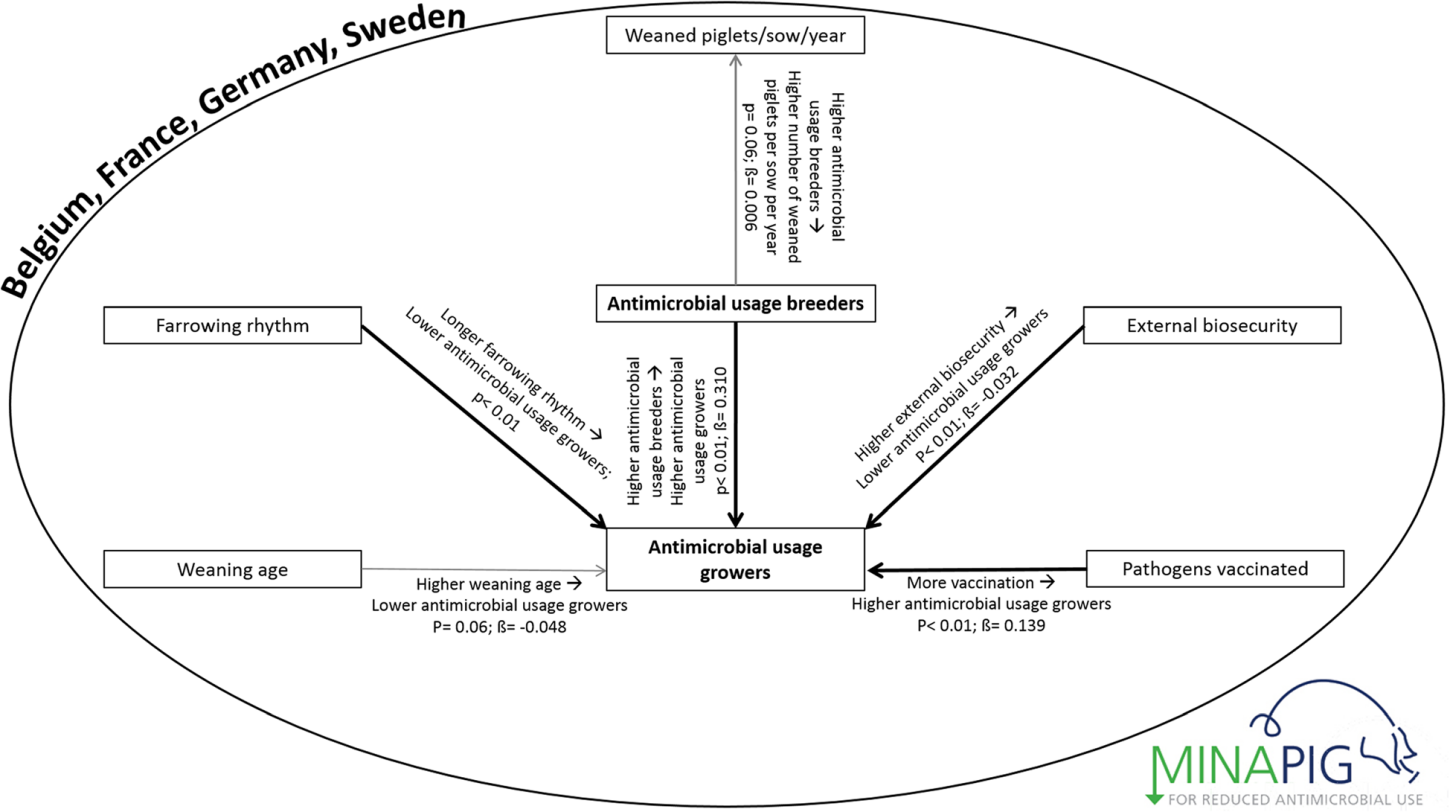
Causal pathway associations for TI 200 days and TI Breeding. Causal pathway with statistically significant associations in the multivariable models for the TI 200 days and the TI Breeding associated with production, management or biosecurity variables. TI = treatment incidence (antimicrobial usage quantification), WSY = number of weaned piglets per sow per year. Black lines represent the result of a multivariable linear regression analysis based on data from 4 EU countries. The light gray line indicates 0.05 < *p* < 0.10. The p-values and β-values correspond to the multivariable model. All models were corrected for the country effect by placing country as a fixed variable in the model, hence the circle around the figure

The country corrected univariable analysis resulted in retaining several variables related with the antimicrobial usage or with each other (Table 1). The associations that remained significant in the multivariable models are shown in the causal path in Fig. 1.

The multivariable model for the LOG TI Breeding, corrected for the country effect showed significant associations with the LOG TI 200 (*p* < 0.01). A higher antimicrobial usage in the breeders was associated with a higher antimicrobial usage in the growing pigs (LOG TI 200).

The LOG TI Breeders was positively associated with the number of WSY (*p* = 0.06), meaning that farms with a higher antimicrobial usage in the breeding animals on average weaned slightly more piglets, however, the ß-value was low.

For the LOG TI 200 the multivariable model showed, after correction for a possible country effect, three variables that were directly associated with the antimicrobial usage from birth until slaughter and one that was nearly significant.

The LOG TI 200 was associated with the weaning age (*p* = 0.06); herds with a higher weaning age showed a lower TI 200. A significant (*p* < 0.01) lower TI 200 was observed for 5-week or >5-week systems in comparison to 1-week system. Also for 2-, 3- and 4-week systems a non-significant trend towards a lower TI 200 was observed in comparison to a one week system. Herds with a higher score on their external biosecurity status also showed a lower TI 200 (*p* < 0.01). While herds vaccinating against more pathogens showed a higher TI 200 (*p* < 0.01).

It should be noted that parameters such as the internal biosecurity level, number of sows or employees, gender of the responsible person in the farrowing unit, the education level of the responsible person or the use of products like zinc oxide were not retained in any of the multivariable models associated with antimicrobial usage. The level of antimicrobial usage furthermore was not significantly associated with production parameters such as the ADG or the mortality until weaning.

## Discussion

By showing associations between a higher level of biosecurity, a longer farrowing rhythm or weaning at an older age and a reduced antimicrobial usage the aim of this study was met and the results of this paper have the potential to advise on best practices.

### Associations between antimicrobial usage, biosecurity level and farm characteristics

To overcome national differences in cultures, habits, regulations, pig production structure, disease prevalence and other external factors all models were corrected for country by adding country as a fixed factor in all models. The obtained association are therefore corrected for the country effect and interaction with country was tested as well.

In Fig. 1 the causal pathway shows an association (*p* = 0.06) between the TI Breeding and the number of weaned piglets (WSY). Improved piglet survival might be the result of a more active presence in the farrowing unit by the farmer during the farrowing period, which is the most likely period of antimicrobial treatment for sows. When better attention is paid during the farrowing process piglet survival might improve, resulting in more weaned piglets per sow per year [30, 31]. Another possible explanation for the positive association between the TI Breeding and the WSY could be the increase in the farrowing index due to a positive effect of the antimicrobial usage in the reduced incidence of mastitis and endometritis problems in the sows. A healthy sow might also nurse her piglets better, resulting in a more optimal transmission of maternal antibodies. Although we assumed in the causal pathway that treatment of breeders could have an effect on the number of weaned piglets per sow per year, it may also be possible that in fact the association could be reversed and that high productive sows are more sensitive to diseases and require more antimicrobial treatments, in which case the higher productivity would lead to a higher TI Breeding. Other unmeasured factors might have also influenced this outcome. In all cases we should keep in mind that the association we found was only minor, with a low ß-coefficient and should therefore not be used as an excuse to increase antimicrobial usage in breeding animals. Furthermore, antimicrobial usage in the sow was recently negative associated with the bacterial gut flora and antimicrobial resistance levels of the piglet [32].

The link between the level of usage in the breeding animals with the level of usage in pigs from birth to slaughter was expected, as a high overall disease pressure in a herd may explain the high usage in both breeding animals and the animals from birth until slaughter. A limited number of herds concentrated the majority of antimicrobial treatments and a certain attitude/behaviour of the farmer towards regular usage of medicines might be another explanation for the association between usage in breeding animals and growers [33].

Vaccines are used to improve the immunity status of the animals which should result in a reduced risk for animals to become diseased and subsequently leading to a reduced need for antimicrobial treatment. Therefore vaccines are often suggested as a suitable alternative for antimicrobial use [10, 12, 34–37]. This is an apparent contradiction with the observed positive association between the number of pathogens vaccinated against and the TI 200 in the present study, although this was also observed by Temtem et al. [38]. This association might be due to a high disease pressure on these herds which has not (yet) been brought under control through vaccination, due to insufficient detection of disease, or it might again be an indication of a certain attitude of the farmer and/or his veterinarian, i.e. using/prescribing a lot of veterinary medicinal products as an insurance against disease [33, 39]. This association could be further explored by looking at vaccination details, disease pressure and antimicrobial treatment indications.

The association we found between the weaning age (*p* = 0.06) and TI 200 suggests that a higher weaning age results in healthier, more robust animals who have a reduced necessity for antimicrobials. This is in agreement with the idea that stronger animals, for example when weaned at a later age, are also more likely to have better coping abilities against possible (pathogenic) threats [40, 41].

For the farrowing rhythm we found that a 5-week and ≥ 5-week system were significantly associated with a lower antimicrobial usage. Also for 2-, 3- and 4-week systems a non-significant trend towards a lower TI 200 was observed in comparison to a one week system. We did furthermore see that the herds with a 3-week system had on average a higher weaning age, while for example a 4-week system had lower average weaning ages in Belgium, France and Germany [19]. However, since both variables were included in the multivariable model this indirect effect was already accounted for and the single effects of the farrowing rhythm and the weaning age on the TI 200 both hold strong. One explanation for the lower TI 200 in longer farrowing rhythms might be that a longer period in-between two batches guarantees a better separation between the age groups and allows for more cleaning and disinfection time, resulting in less risk of transmission of pathogens between them. For example Nathues et al. [42] showed that piglets within a herd with a 3-week system were less likely to be infected with *Mycoplasma hyopneumoniae* compared to a 2-week system. His findings did not hold true for a 4-week system, but more pathogens and factors most likely influence our finding and not only *M. hyopneumoniae*, resulting in a positive effect in general for the longer farrowing rhythms.

Both the better results related to a longer farrowing rhythm and even more important the finding that a higher weaning age lead to a lower TI 200 might be of great relevance in future advising of pig farmers to reduce their antimicrobial usage. Further research should investigate this association in more detail and determine whether this trend can be confirmed. If so, it would be possible to reduce antimicrobial usage by developing more strict regulation and legislation on the weaning age.

A last important finding was the association between the level of external biosecurity and the TI 200 (*p* < 0.01). External biosecurity controls the risk of entrance and exit of pathogens into a herd. Introduction of pathogens from an outside source poses the largest risk for disease onset in pig production [24, 43–45]. When we would be able to reduce this risk it is also likely that less antimicrobials would be needed [46, 47]. Moreover, external and internal biosecurity are shown to be highly correlated to each other [19]. Due to this association internal biosecurity improvement might also have an effect on the antimicrobial usage from birth until slaughter. Laanen et al. [20] showed this association between internal biosecurity and TI in her study performed in Belgium in 2009–2010. Since improvement of the internal biosecurity level could be a rather simple intervention (e.g. strict hygiene protocols, correct use of working lines) at herd level, this might be an important consideration in the reduction of antimicrobial usage.

We should also stress that no significant positive associations were found between a higher usage and better production results such as ADG or mortality, as also supported by a paper of van der Fels-Klerx et al. [48]. Although it is sometimes suggested that the use of antimicrobials results in heavier pigs, as also stimulated in earlier years by the use of antimicrobial growth promotors in the feed, we did not find a significant link in this study. In the EU the use of antimicrobial growth promotors in feed was banned since 2006 [49]. The use of zinc oxide also showed no association with antimicrobial usage, although often it is promoted as an alternative in the reduction of antimicrobial usage [12, 50]. Unknown however, was how long the herds already used zinc oxide and whether this could already have affected their antimicrobial usage. Improved health of the pigs might result in a better ADG and lower mortality, however, no significant direct association between these and the antimicrobial usage were found and most likely more factors were of importance in the herds’ ADG and mortality results. Results suggest that administering antimicrobials did not improve technical results.

Future studies should try to confirm the above presented findings so that they could be validated as successful actions in the reduction of antimicrobial usage.

### Study design and limitations

Only a limited number of studies have investigated the associations between production parameters, other herd characteristics and antimicrobial usage [20]. A recent review of Aarestrup [10] emphasizes the need for research on effects of interventions. The current study attempts to provide a first overview of the associations between production parameters, preventive measures such as high biosecurity status and vaccination level and herd and management characteristics with the level of antimicrobial usage. Knowledge on these associations might be used as input for future intervention studies.

We should however, be aware that this study is likely influenced by the fact that the participating farmers resembled the better end of the population since their selection was based on willingness to participate (except in France where random sampling was used, by selecting herds from the database of Institute for pig and pork industry including on average 53 % of herds located in North-West France with >49 sows) and interest in the topic, resulting in a selection bias. Variability between researchers was minimized by providing all interviewers with the same training in execution of the questionnaire form, however, it might have caused some random noise as well. In France information on antimicrobial usage was collected from the last batch whereas for the other countries the year preceding the herd visit was used. This could have led to a limited bias due to difference in disease prevalence in combination with seasonal influences. Recall bias was considered to be of minimal importance since the majority of collected information was checked using visual inspection and/or documentation. We should also stress that the obtained associations were the result of a cross-sectional study design, not allowing to make direct causal conclusions. By designing the causal pathway however, we tried to give a clear overview of obtained associations.

## Conclusions

This cross-sectional study on 227 pig herds in Belgium, France, Germany and Sweden showed that the antimicrobial usage in breeding animals tends to be positively associated with the number of weaned piglets per sow per year and the antimicrobial usage from birth to slaughter (TI 200) in growing pigs. The TI 200 was shown to be lower in herds with a farrowing rhythm ≥5-weeks, a higher biosecurity status and tended to be lower with weaning of the piglets at an older age. Policy makers, herd advisors and farmers should benefit from this knowledge in order to reduce the antimicrobial usage on pig herds.

## Abbreviations

ADG: average daily weight gain
AM: antimicrobial
AMR: antimicrobial resistance
AMU: antimicrobial usage
DDDA: defined daily dose animal
EU: European Union
LOG: logaritmic
TI: treatment incidence
WSY: number of weaned piglets per sow per year
